# Dysfunctional Noise Cancelling of the Rostral Anterior Cingulate Cortex in Tinnitus Patients

**DOI:** 10.1371/journal.pone.0123538

**Published:** 2015-04-13

**Authors:** Jae Jin Song, Sven Vanneste, Dirk De Ridder

**Affiliations:** 1 Department of Otorhinolaryngology-Head and Neck Surgery, Seoul National University Bundang Hospital, Seongnam, Korea; 2 Department of Translational Neuroscience, Faculty of Medicine, University of Antwerp, Edegem, Belgium; 3 Lab for Auditory and Integrative Neuroscience, School of Behavioral and Brain Sciences, The University of Texas at Dallas, Dallas, United States of America; 4 Unit of Neurosurgery, Department of Surgical Sciences, Dunedin School of Medicine, University of Otago, Dunedin, New Zealand; 5 BRAI^2^N, Sint Augustinus Hospital, Antwerp, Belgium; University of Salamanca- Institute for Neuroscience of Castille and Leon and Medical School, SPAIN

## Abstract

**Background:**

Peripheral auditory deafferentation and central compensation have been regarded as the main culprits of tinnitus generation. However, patient-to-patient discrepancy in the range of the percentage of daytime in which tinnitus is perceived (tinnitus awareness percentage, 0 – 100%), is not fully explicable only by peripheral deafferentation, considering that the deafferentation is a stable persisting phenomenon but tinnitus is intermittently perceived in most patients. Consequently, the involvement of a dysfunctional noise cancellation mechanism has recently been suggested with regard to the individual differences in reported tinnitus awareness. By correlating the tinnitus awareness percentage with resting-state source-localized electroencephalography findings, we may be able to retrieve the cortical area that is negatively correlated with tinnitus awareness percentage, and then the area may be regarded as the core of the noise cancelling system that is defective in patients with tinnitus.

**Methods and Findings:**

Using resting-state cortical oscillation, we investigated 80 tinnitus patients by correlating the tinnitus awareness percentage with their source-localized cortical oscillatory activity and functional connectivity. The activity of bilateral rostral anterior cingulate cortices (ACCs), left dorsal- and pregenual ACCs for the delta band, bilateral rostral/pregenual/subgenual ACCs for the theta band, and left rostral/pregenual ACC for the beta 1 band displayed significantly negative correlations with tinnitus awareness percentage. Also, the connectivity between the left primary auditory cortex (A1) and the rostral ACC, as well as between the left A1 and the subgenual ACC for the beta 1 band, were negatively correlated with tinnitus awareness percentage.

**Conclusions:**

These results may designate the role of the rostral ACC as the core of the descending noise cancellation system, and thus dysfunction of the rostral ACC may result in perception of tinnitus. The present study also opens a possibility of tinnitus modulation by neuromodulatory approaches targeting the rostral ACC.

## Introduction

Tinnitus, the perception of internal sound without an external source, develops in 15–20% of adults at some point in their lifetime and interferes severely with the quality of life in 5–26% of the afflicted population [1,2]. However, the underlying pathophysiologic mechanism of non-pulsatile subjective tinnitus, the most common type of tinnitus, is poorly understood despite its relatively high prevalence and simple manifestation. Non-pulsatile tinnitus is frequently associated with auditory deafferentation in cases of sensorineural hearing loss [3–5], a notion supported by transient phantom sound perception after experimentally induced partial and complete auditory deprivation in normal subjects [6,7]. Previous researchers have suggested the auditory deafferentation and resultant compensatory changes in the central auditory system as the main culprit of tinnitus generation, and thus an up-regulation of spontaneous firing rates [8], tonotopic map reorganization and increased neural synchrony [9], increased central noise [10], synchronous neuronal activity of cell assemblies within the auditory cortex [11], and a loss of lateral inhibition [12] have been proposed to be associated with tinnitus generation.

Nevertheless, tinnitus perception is not entirely explainable by the changes in the central auditory system in that only a subset of hearing loss accompanies tinnitus [13] and neuroimaging studies have consistently shown limbic system involvement in tinnitus [14–17]. Based on these observations, a “dysfunctional noise cancelling mechanism” has recently been conceptualized [18,19]. According to this concept, patients become aware of tinnitus only if the noise (tinnitus) cancellation system fails to suppress the tinnitus signal generated by auditory cortical changes. For the noise cancellation system, the ventromedial prefrontal cortex (vmPFC) [18] has been suggested to be one of the core regions, and this was confirmed by structural [20] and functional [21] imaging studies in patients with chronic tinnitus, but other structural imaging studies failed to find vmPFC as the core region [22,23]. Meanwhile, because fluctuations of activity in the anterior cingulate cortex (ACC) and anterior insula determine whether a near threshold pain stimulus is consciously perceived or not [24], the ACC and anterior insula, also known as the components of “salience network” that relate to interoceptive-autonomic processing [25], have been suggested to be another core network for the noise cancelling system, based on the similarity of pain and tinnitus pathways [19,26].

The similarities of the symptomatology (i.e. phantom percepts of sensory stimuli), as well as pathogenesis between tinnitus and phantom pain, have already been noted by previous authors [26–28] and have been subsumed under the term "maladaptive plasticity diseases" [29]. For pain, at least two ascending and one descending pathways have been described. The ascending system consists of a medial and lateral pathway, linked to the sensory discriminative and affective attentional components of the pain [30]. The sensory component has been proposed to be mediated by a lateral pain system comprised of the thalamic ventroposterolateral nucleus, primary and secondary somatosensory cortex, parietal cortex, and the affective component by a medial pain system comprised of the thalamic dorsomedial nucleus, amygdala, dorsal ACC, and insula [30–33]. Recently, a possible existence of a medial auditory processing system has been suggested [19] based on the existence of auditory processing cells in the thalamic dorsomedial nucleus [34] and the involvement of the amygdala, dorsal ACC, and insula in processing an affective component of sound [35–38]. Not only have ascending pathways been described, but also descending inhibitory systems for pain [39–41], and the descending pain inhibitory system involves medial brain areas such as the anterior insula, pregenual ACC, and periaqueductal gray [39–41]. Analogous to this descending pain inhibitory system, a similar noise cancellation system has been suggested in tinnitus by different authors [18,19].

However, these candidate core regions have never been evaluated by measuring resting-state cortical electrical brain activity and functional connectivity. Given that tinnitus is an internally generated sound perception, even though there is no external sound source, it is essential to conduct a study exploring cortical areas that are responsible for the dysfunctional noise cancelling system under resting state with minimal sound input. By correlating the percentage of daytime in which tinnitus is perceived (tinnitus awareness percentage) with resting-state source-localized electroencephalography (EEG) findings, we may be able to retrieve the cortical area that is negatively correlated with tinnitus awareness percentage. Then the area may be regarded as the core of the noise cancelling system that is defective in patients with tinnitus. The underlying idea is that the more defective the noise cancelling mechanism is, the more time during the day that the tinnitus will be perceived, considering that while deafferentation is involved in tinnitus, variability in tinnitus awareness over time or between individuals likely involves additional factors. Additionally, by performing lagged linear connectivity studies, we sought to reveal connectivity changes in the putative noise cancelling system in patients with chronic tinnitus.

## Materials and Methods

### Participants and questionnaire

To maintain a homogenous study group and to minimize cortical activity changes due to tinnitus laterality and characteristics [42,43], we selected a total of 450 participants with bilateral (“almost equal loudness on both ears” according to the patients’ description) narrow band noise (NBN) tinnitus from the database of the multidisciplinary Tinnitus Research Initiative Clinic of the University Hospital of Antwerp, Belgium. Of them, in order to control for cortical activity bias by hearing loss, individuals with a hearing threshold exceeding 20 dB HL, as measured by a conventional hearing threshold calculation method (mean value of hearing thresholds at 0.5, 1, and 2 kHz) [44,45], in at least one ear were excluded from the study. Also, individuals with pulsatile- or objective tinnitus, otologic disorders such as Ménière's disease or otosclerosis, psychiatric or neurological disorders, chronic headache, drug or alcohol abuse, current psychotropic or central nervous system-active medications, history of head injury (with loss of consciousness) or seizures were not included in the study. In this way, 80 of 450 participants with bilateral NBN tinnitus (54 males and 26 females) with a mean age of 47.2 ±13.9 years (range, 20–76) were finally included in the study. Antwerp University Hospital Ethics Committee reviewed and approved the study and all applicable documents prior to study initiation. All patients signed an approved informed consent in order to be enrolled into the study.

All selected participants answered a validated Dutch version of the tinnitus questionnaire (TQ) [46,47], Numeric Rating Scale (NRS) of tinnitus intensity (answering to a question “how loud is your tinnitus?” on a scale from 0 to 10) and tinnitus-related distress (answering to a question “how bothered are you by your tinnitus?” on a scale from 0 to 10), percentage of daytime during which the participant is distressed by tinnitus (distressed time percentage), and percentage of daytime during which the participant is aware of the tinnitus (tinnitus awareness percentage). All participants underwent audiometry to measure hearing threshold and tinnitus matching to evaluate tinnitus frequency and intensity. All the variables, TQ (36.0 ± 14.1), NRS intensity (5.1 ± 2.3), NRS distress (4.8 ± 2.5), distressed time percentage (52.5 ± 31.3%), and tinnitus awareness percentage (63.5 ± 27.5%), were normally distributed. The characteristics and questionnaire scores of the 80 participants are listed in Table 1.

**Table 1:** The characteristics and questionnaire scores of the 80 participants.

patient number	age (years)	sex	TQ	NRS intensity	NRS distress	TAP (%)	DTP (%)
1	46	M	19	7	7	80	35
2	41	M	52	9	8	90	80
3	31	F	51	3	3	75	0
4	66	M	28	6	8	50	50
5	58	M	52	7	8	100	100
6	36	M	49	3	3	30	30
7	42	M	45	6	6	55	50
8	57	M	20	2	2	60	40
9	27	M	33	1	2	100	10
10	45	M	34	4	2	100	50
11	50	F	39	8	5	50	30
12	62	M	33	6	6	30	25
13	29	M	25	1	0	5	5
14	66	F	44	5	4	50	50
15	49	M	37	7	6	100	70
16	65	M	61	4	4	50	100
17	23	M	58	5	8	25	10
18	29	M	20	4	4	70	50
19	29	F	52	8	8	80	70
20	43	M	41	5	5	100	90
21	51	M	36	5	8	95	95
22	28	F	42	5	5	30	15
23	35	M	24	2	2	10	10
24	41	M	48	2	3	65	75
25	46	M	33	8	4	75	35
26	20	F	25	2	2	30	75
27	36	M	36	4	4	50	35
28	63	F	40	2	1	10	10
29	24	M	42	5	4	80	80
30	54	M	30	4	3	25	20
31	35	M	21	7	4	15	15
32	64	M	42	6	7	65	65
33	41	F	49	5	5	50	50
34	70	F	47	8	8	100	100
35	48	M	20	2	6	50	10
36	60	M	39	8	8	100	100
37	48	F	11	3	0	25	10
38	41	M	66	7	8	100	100
39	37	F	18	3	5	50	25
40	33	M	46	7	4	75	75
41	46	M	18	1.5	2	25	5
42	30	F	48	6	6	60	40
43	45	M	29	5	5	50	30
44	53	F	8	3	3	30	30
45	29	M	50	5	5	50	60
46	49	M	29	4	4	75	50
47	59	M	49	4	3	90	60
48	67	M	50	4	4	70	50
49	42	M	38	7	7	80	50
50	50	M	26	7	7	70	70
51	65	M	51	8.5	8.5	100	80
52	20	M	50	8	9	80	90
53	53	F	14	6	3	70	25
54	54	M	18	4	2	90	90
55	62	F	21	3	3	50	50
56	30	M	15	6	4	75	50
57	72	F	38	7	7	75	70
58	43	F	34	7	6	77.5	65
59	65	M	42	6	8	90	90
60	52	F	23	6	6	15	15
61	58	M	60	7	6	90	70
62	46	F	52	2	2	94	94
63	43	F	18	4	0	50	20
64	39	F	70	9	10	97.5	97.5
65	52	M	25	4	6	30	15
66	54	F	42	7	7	100	100
67	54	F	13	5	2	20	20
68	76	M	29	5	4	90	60
69	59	F	58	7	7	100	100
70	29	M	37	5	6	75	100
71	63	M	28	7	5	40	30
72	69	M	24	8	7	60	5
73	36	F	25	2	0	50	80
74	27	M	43	5	8	35	35
75	55	F	33	7	7	60	30
76	63	M	35	7	7	70	70
77	54	F	27	8	4	80	75
78	52	M	44	8	6	100	100
79	68	M	13	7	6	30	20
80	53	M	47	7	8	85	85

TQ, tinnitus questionnaire; NRS, numeric rating scale; TAP, tinnitus awareness percentage; DTP, distressed time percentage; M, male; F, female.

In order to identify possible affecting factors of the tinnitus awareness percentage, Pearson correlations were calculated between the tinnitus awareness percentage and other parameters such as time distress percentage, TQ score, NRS tinnitus intensity, age, and the duration of tinnitus.

### EEG recording

EEGs were recorded for 5 minutes at 19 scalp sites of a Tin-electrode cap (ElectroCap, Ohio, United States), using a Mitsar amplifier (Mitsar EEG-201, St.Petersburg, Russia) and the WinEEG software version 2.84.44 (Mitsar, St. Pertersburg, Russia; http://www.mitsar-medical.com) in a fully lighted room shielded from sound and stray electric fields, with each participant's eyes closed and sitting upright in a comfortable chair. The EEG was sampled with 19 electrodes in the standard 10–20 International placement referenced to linked ears and impedances were maintained below 5 kΩ at all electrodes throughout the EEG recording. Data were recorded with a sampling rate of 1024 Hz, a high-pass filter of 0.15 Hz, and a low-pass filter of 200 Hz. The raw data were initially processed by resampling to 128 Hz and band-pass filtering (fast Fourier transform filter applying a Hanning window) with 2–44 Hz, imported into the Eureka! software [48], then plotted and carefully inspected for manual artifact-rejection. A careful inspection of artifacts was performed and all episodic artifacts including eye blinks, eye movements, teeth clenching, or body movement were manually removed from the EEG stream.

Participants were instructed not to drink alcohol 24 hours prior to EEG recording and to abstain from caffeinated beverages on the day of recording to avoid alcohol-induced changes in EEG [49] or a caffeine-induced alpha and beta power decrease [50,51]. The vigilance of participants was checked by monitoring EEG streams on the screen such as slowing of the alpha rhythm or appearance of spindles to prevent possible enhancement of the theta power due to drowsiness [52], and no participants included in the current study showed such drowsiness-related EEG changes.

### Source localization analysis

Standardized low-resolution brain electromagnetic tomography (sLORETA, available at http://www.unizh.ch/keyinst/NewLORETA/LORETA01.htm), a functional imaging method yielding standardized current density based on certain electrophysiological and neuroanatomical constraints [53], was utilized to estimate the intracerebral sources generating the scalp-recorded electrical activity in each of the following eight frequency bands: delta (2–3.5 Hz), theta (4–7.5 Hz), alpha1 (8–10 Hz), alpha2 (10–12 Hz), beta1 (13–18 Hz), beta2 (18.5–21 Hz), beta3 (21.5–30 Hz), and gamma (30.5–44 Hz) [17,54–57]. Because the sLORETA itself corrects for multiple testing (i.e., for the collection of tests performed for all electrodes and/or voxels [53,58], and for all time samples and/or discrete frequencies), no further statistical correction is required for multiple comparison. The sLORETA algorithm solves the inverse problem, the computation of images of electric neuronal activity based on extracranial measurements, by assuming related orientations and strengths of neighboring neuronal sources that are represented by adjacent voxels. The solution space used in this study and associated lead field matrix are those implemented in the LORETA-Key software (available at http://www.uzh.ch/keyinst/loreta.htm). This software implements revisited realistic electrode coordinates [59] and the lead field produced by Fuchs et al [60] applying the boundary element method on the MNI-152 (Montreal neurological institute, Canada). The sLORETA-key anatomical template divides and labels the neocortical (including the hippocampus and ACC) MNI-152 volume in 6,239 voxels with a size of 5 x 5 x 5 mm, based on probabilities returned by the Daemon Atlas (Lancaster et al. 2000). The co-registration makes use of the correct translation from the MNI-152 space into the Talairach and Tournoux space. Anatomical labelling of significant clusters was done by sLORETA built-in toolbox.

### Functional connectivity analysis

Coherence and phase synchronization between time series corresponding to different regions of interest (ROIs) were calculated to analyze functional connectivity using the connectivity toolbox in sLORETA. In the connectivity toolbox of sLORETA, measures of linear dependence (coherence) and nonlinear dependence (phase synchronization) between multivariate time series are defined and the measures are expressed as the sum of lagged/instantaneous dependence. The measures are non-negative, and take the value zero only when there is independence of the pertinent type (lagged, instantaneous, or both) and they are defined in the above-described eight frequency bands. For functional connectivity analysis, a total of 20 Brodmann areas, encompassing ROIs such as bilateral auditory cortices [61–63], bilateral parahippocampus [23], bilateral rostral/pregenual/subgenual ACCs [38,64,65], and bilateral vmPFC [20,66], were selected as possible nodes based on previous literature (see Table 2).

**Table 2:** Twenty regions of interest (0.125 cm^3^ each), their center-of-mass coordinates, references.

**Regions of interest**	**BA**	**Center-of mass coordinates**			**References**
		x	y	z	
**Auditory cortices**	41L	-46	-29	10	[61–63]
	41R	47	-29	10	
	42L	-62	-23	12	
	42R	63	-24	12	
	21L	-57	-18	-15	
	21R	58	-17	-15	
	22L	-56	-25	5	
	22R	56	-22	3	
**Rostral anterior cingulate cortex**	24L	-8	2	36	[64,65]
	24R	7	1	36	
**Pregenual anterior cingulate cortex**	32L	-9	29	21	[64]
	32R	8	30	20	
**Subgenual anterior cingulate cortex**	25L	-8	18	-17	[38]
	25R	5	14	-14	
**Parahippocampus**	27L	-19	-33	-4	[23]
	27R	18	-33	-4	
	29L	-7	-50	7	
	29R	6	-50	8	
**Ventromedial prefrontal cortex**	10L	-22	54	9	[20,66]
	10R	22	54	9	

BA, Brodmann area; L, left; R, right.

Coordinates are described in MNI coordinates.

### Statistical analyses

The statistical analysis method used for the source localization and connectivity analyses is statistical non-parametric mapping (SnPM) using a permutation test on the labels for comparison. The SnPM accounts for the multiple comparisons problem implicit in the standard voxel-by-voxel hypothesis testing framework and gives results similar to those obtained from a comparable Statistical Parametric Mapping approach using a general linear model with multiple comparisons corrections derived from random field theory [58]. Namely, the SnPM corrects for multiple tests performed for all voxels, and for all frequency bands. Due to the non-parametric nature of this method, the validity of the SnPM does not rely on any assumption of Gaussianity [58]. The significance threshold for all tests was based on a permutation test with 5,000 permutations.

An sLORETA correlation analysis testing the statistical correlation between tinnitus awareness percentage and voxel-by-voxel current density distribution for the 8 different frequency bands was performed to find areas that are significantly correlated with tinnitus perception using sLORETA’s built-in voxelwise randomization tests (5000 permutations), with a threshold *P* < .05. A correction for multiple comparisons in SnPM, using random permutations (5,000 permutations in the current study), has been validated and proven to give results similar to or better than those obtained from a comparable statistical parametric mapping approach using a general linear model with multiple comparisons corrections derived from random field theory [58,67]. In the same way, for lagged linear connectivity, a regression analysis was performed to find significant correlations between tinnitus awareness percentage and lagged linear connectivity among 20 ROIs for the 8 frequency bands employing the t-statistics s with a corrected threshold *P* < .05. The significance threshold was corrected for multiple comparisons by conducting sLORETA-built-in voxelwise randomization tests (5,000 permutations).

### Region of interest analysis

The log-transformed electric current density was averaged across all voxels belonging to the regions that displayed significant results on source localization and functional connectivity analyses. For these regions, first simple Pearson’s correlation analyses then partial correlation analyses were performed to control for all the factors (time distress percentage, age, TQ score, NRS tinnitus intensity, NRS tinnitus distress, and duration of tinnitus) that were investigated for a correlation with tinnitus awareness percentage. For partial correlation analyses, the controlled factors were kept aligned with the tinnitus awareness percentage data. In this way, we could measure the degree of correlation between the selected regions and tinnitus awareness percentage, while removing the effect of control variables. The statistical significance was set at *P* < .05 after Bonferroni correction for multiple comparisons by multiplying the uncorrected *P* value by the number of frequency bands. To further confirm the areas that were significantly correlated with tinnitus awareness percentage by partial correlation analyses, a multivariate analysis of variance (MANOVA, i.e. Wilks’ Lambda) was performed with these significantly correlated areas as dependent variables and all the factors used for the partial correlation analysis (including tinnitus awareness percentage itself) as independent variables. The effect size of independent variables was determined by comparing MANOVA’s eta squared (η^2^). All statistical analyses were conducted by SPSS (version 20.0, SPSS Inc., Chicago, IL).

**Fig 1:**
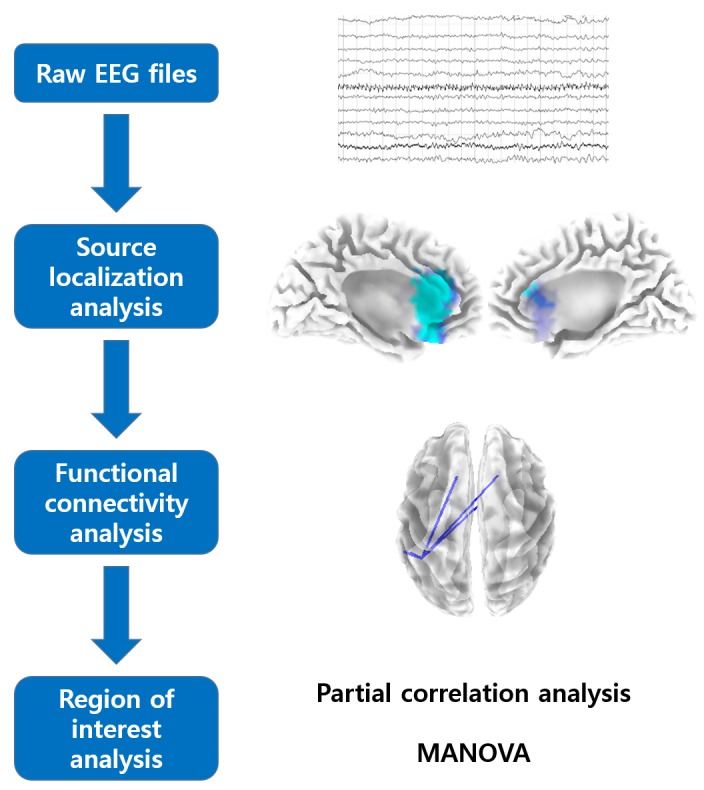
The analysis pipeline of the current study.

Fig 1 depicts the analysis pipeline summarizing three different methods used for the current study.

## Results

### Correlation analysis between tinnitus awareness percentage and other parameters

Pearson correlation analysis revealed that tinnitus awareness percentage correlates positively with distressed time percentage (Pearson’s r = .75, *P* < .01), TQ score (r = .48, *P* < .01), NRS tinnitus intensity (r = .41, *P* < .01), NRS tinnitus distress (r = .39, *P* < .01), and the duration of tinnitus (r = .26, *P* = .02). By contrast, the age of tinnitus subjects showed no significant correlation with tinnitus awareness percentage (r = .15, *P* = .19).

### Source-localized correlation analysis

**Fig 2:**
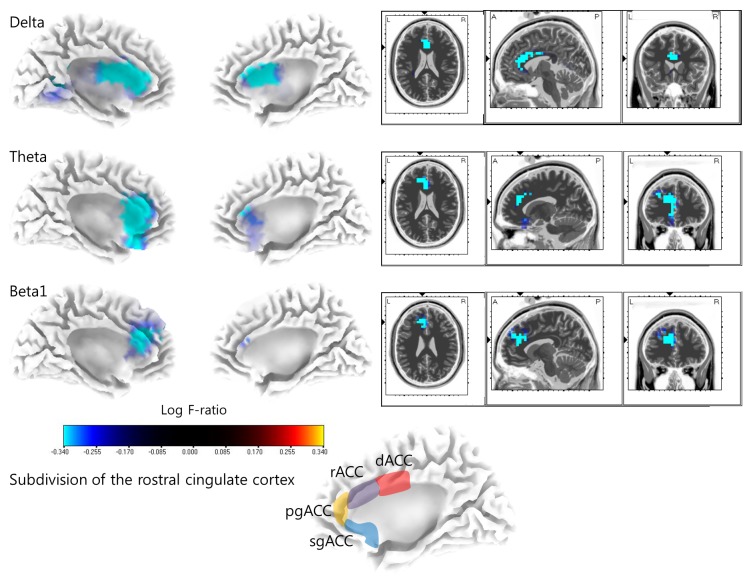
Standardized low-resolution brain electromagnetic tomography (sLORETA) correlation analysis with tinnitus awareness percentage. The activity of bilateral rostral anterior cingulate cortices (rostral ACCs), left dorsal anterior cingulate cortex (dACC), and left pregenual anterior cingulate cortex (pgACC) for the delta band, bilateral rostral ACCs, pgACCs, and subgenual anterior cingulate cortices (sgACCs, BA 25) for the theta band, and left rostral ACC and pgACC for the beta 1 band displayed significantly negative correlations with tinnitus awareness percentage. The anatomical subdivisions of the ACC are illustrated in the lowermost panel (modified from [68]).

The activity of the bilateral rostral ACCs, left dorsal ACC, and left pregenual ACC for the delta band, bilateral rostral ACCs, pregenual ACCs, and subgenual ACCs for the theta band, and left rostral ACC and pregenual ACC for the beta 1 band displayed significantly negative correlations (*P* < .05) with tinnitus awareness percentage (Fig 2). By contrast, no significant positive- or negative correlations were found for the alpha 1 and 2, beta 2 and 3, and gamma bands. Meanwhile, the oscillatory brain activity showed no significant positive- or negative correlations with distressed time percentage, TQ score, NRS tinnitus intensity, or NRS tinnitus distress.

### Functional connectivity-based correlation analysis

**Fig 3:**
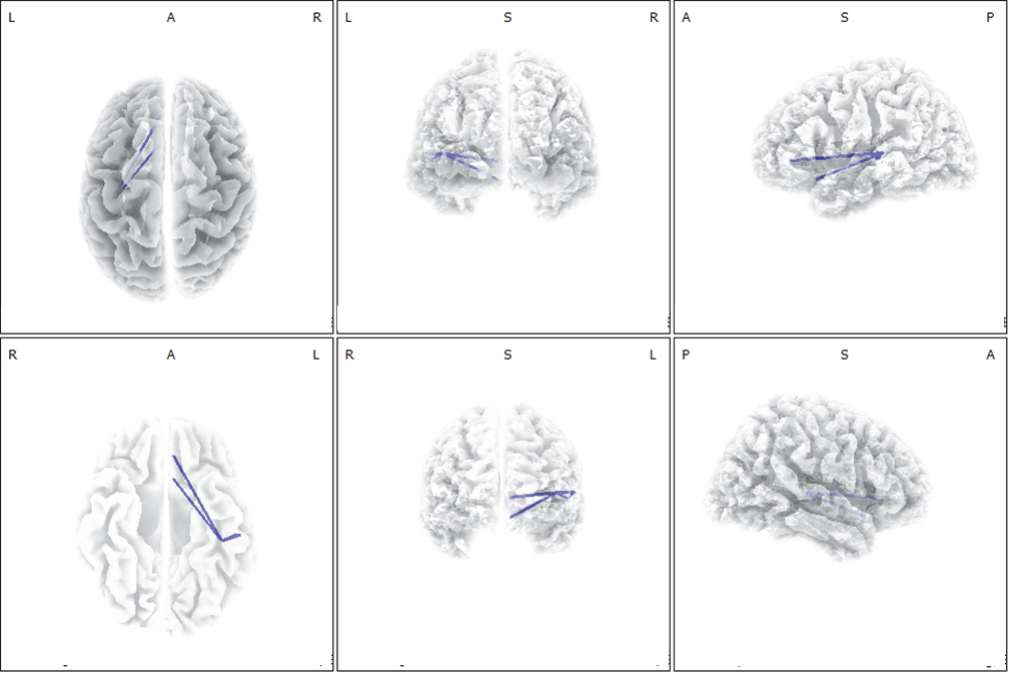
Functional connectivity-based correlation analysis with tinnitus awareness percentage. The lagged linear connectivity between the left primary auditory cortex (A1) and the rostral anterior cingulate cortex as well as between the left A1 and the subgenual anterior cingulate cortex for the beta 1 band were negatively correlated with tinnitus awareness percentage.

The lagged linear connectivity between the left primary auditory cortex (A1, BA 41) and the rostral ACC, as well as between the left A1 and the subgenual ACC for the beta 1 band, were negatively correlated with tinnitus awareness percentage (*P* < .05, Fig 3). For the other seven frequency bands, no significant correlations between the lagged linear connectivity and tinnitus awareness percentage were found.

### ROI analysis: partial correlation analysis and MANOVA

The average log-transformed electric current density of the ROIs that showed significantly negative correlations by the source localization and functional connectivity analyses (the rostral/pregenual ACC for the delta band, the rostral/pregenual/subgenual ACC for the theta band, and rostral ACC/pregenual ACC for the beta 1 band) showed all significant correlations with tinnitus awareness percentage both by simple Pearson’s correlation analysis and partial correlation analysis controlling for time distress percentage, TQ score, NRS tinnitus intensity, age, and the duration of tinnitus, using a threshold of uncorrected *P* < .05. Of these 7 ROIs, the rostral ACC for the delta band and the rostral/pregenual ACC for the beta 1 band still retained significantly negative correlations with tinnitus awareness percentage after Bonferroni correction for multiple comparisons (Table 3). In other words, the rostral ACC for the delta band and the rostral/pregenual ACC for the beta 1 band showed negative correlations with the tinnitus awareness percentage even after removing all the effect of control variables.

**Table 3:** Significant partial correlations between tinnitus awareness percentage and the average log-transformed electric current density of the regions of interest controlled for time distress percentage, tinnitus questionnaire score, age, and the duration of tinnitus.

		**Correlation coefficient**	**Partial correlation coefficient**	**Uncorrected *P***	**Corrected *P* for multiple comparison**	**df**
**Delta**	**rACC**	-.337	-.331	.004	.033*	71
	**pgACC**	-.357	-.257	.028	.224	71
**Theta**	**rACC**	-.328	-.286	.014	.115	71
	**pgACC**	-.398	-.301	.010	.077	71
	**sgACC**	-.337	-.247	.035	.283	71
**Beta1**	**rACC**	-.285	-.322	.006	.044*	71
	**pgACC**	-.361	-.346	.003	.022*	71

*Corrected *P* < 0.05.

Df, degree of freedom; rACC, rostral anterior cingulate cortex; pgACC, pregenual anterior cingulate cortex; sgACC, subgenual anterior cingulate cortex.

For these 3 areas of significant partial correlations with tinnitus awareness percentage, MANOVA analysis was performed to confirm the effect size by comparing MANOVA’s η^2^. For the rostral ACC for the delta band, although the age showed significance (*P* = .025) with moderate effect size (η^2^ = .065) according to Cohen’s definition (.01 ≤ η^2^ < .06, a small effect;. 06 ≤ η^2^ < .14, a moderate effect; η^2^ ≥. 14 or higher, a large effect) [69], tinnitus awareness percentage showed the most significant value (*P* = .003) with the largest effect size (η^2^ = .113) (Table 4, upper row). For the rostral ACC for the beta 1 band, tinnitus awareness percentage was the only dependent variable that showed significance (*P* = .012) with moderate effect size (η^2^ = .079) (Table 4, middle row). For the pregenual ACC for the beta 1 band, although the age showed significance (*P* = .001), tinnitus awareness percentage showed the most significant value with the only moderate effect size (η^2^ = .129) of six dependent variables (Table 4, lower row).

**Table 4:** Multivariate analysis of variance with six dependent variables for three regions that showed significant partial correlation with time awareness percentage.

Region of interest	Dependent variables	F	*P* value	η^2^
Delta, rACC	Time awareness percentage	9.638	.003*	.113 ^**†**^
	Time distress percentage	2.374	.127	.030
	Age	5.253	.025*	.065 ^**†**^
	TQ score	.963	.330	.013
	NRS tinnitus intensity	.202	.654	.003
	NRS tinnitus distress	.048	.827	.001
	Duration of tinnitus	2.219	.140	.028
Beta 1, rACC	Time awareness percentage	6.550	.012*	.079 ^**†**^
	Time distress percentage	1.394	.241	.018
	Age	2.045	.157	.026
	TQ score	.003	.959	.000
	NRS tinnitus intensity	.892	.348	.012
	NRS tinnitus distress	.008	.931	.000
	Duration of tinnitus	.431	.514	.006
Beta 1, pgACC	Time awareness percentage	11.256	.001*	.129 ^**†**^
	Time distress percentage	4.170	.045*	.052
	Age	2.049	.156	.026
	TQ score	.435	.512	.006
	NRS tinnitus intensity	.095	.759	.001
	NRS tinnitus distress	.190	.664	.002
	Duration of tinnitus	.015	.901	.000

* *P* < 0.05.

^†^ Moderate effect according to Cohen’s definition [69]

rACC, rostral anterior cingulate cortex; pgACC, pregenual anterior cingulate cortex. TQ, tinnitus questionnaire; NRS, numeric rating scale.

## Discussion

In the current study, we wanted to unravel those areas in the brain that are correlated with the individual differences in reported tinnitus awareness based on the fact that while deafferentation is involved in tinnitus, variability in tinnitus awareness over time or between individuals likely involves additional factors. In summary, the cortical activity of the bilateral rostral ACCs, left dorsal ACC, and left pregenual ACC for the delta band, bilateral dorsal/pregenual/subgenual ACCs for the theta band, and left dorsal/pregenual ACC for the beta 1 band, as well as the lagged linear connectivity between the left A1 and left rostral ACC and between the left A1 and the left subgenual ACC for the beta 1 band, displayed significantly negative correlations with tinnitus awareness percentage. In particular, the rostral ACC for the delta band and the rostral/pregenual ACC for the beta 1 band showed significant correlations, even after controlling for all possible biasing factors by partial correlation and MANOVA analyses on the ROIs that showed the most salient negative correlations on source localization-base correlation analysis. In short, the rostral ACC may be involved the in variability in tinnitus awareness over time or between individuals.

The question is: What is the role of the rostral ACC in the generation or maintenance of the tinnitus percept in consciousness?

### Rostral ACC: the core of the noise cancelling system in tinnitus

The similarities of the symptomatology (i.e. phantom percepts of sensory stimuli) as well as pathogenesis between tinnitus and phantom pain, have already been noted by previous authors [26–28,70]. For pain, at least two ascending and one descending pathways have been described. The ascending system consists of a medial and lateral pathway, linked to the sensory discriminative and affective attentional components of the pain [30]. Recently, a possible existence of a medial auditory processing system has been suggested [19,26] based on the existence of auditory processing cells in the thalamic dorsomedial nucleus [34] and the involvement of the amygdala, dACC, and insula in processing an affective component of sound [35,37,38].

The descending pain inhibitory system involves medial brain areas such as the anterior insula, pgACC, and periaqueductal gray [39–41]. Analogous to this descending pain inhibitory system, a similar noise cancellation system based on limbic-auditory interaction has been suggested in tinnitus [18]. The nucleus accumbens (NAc) of the ventral striatum and the vmPFC have been proposed to be the core regions in this noise cancellation system, and these areas have been supported both by anatomical [20,66] and functional [21,66] MRI studies. In other words, while thalamocortical dysrhythmia (based on diminished excitatory or increased inhibitory input at the thalamic level) [71] or thalamic gating failure (reduced functional connectivity between the inferior colliculi and the A1) [72] assume bottom-up tinnitus generation, dysfunctional noise cancellation system may result in top-down tinnitus generation.

In the current study, source-localized activity in areas of the rostral ACC showed negative correlation with tinnitus awareness percentage, and could therefore be proposed as an indicator of a dysfunctional noise cancellation system. Moreover, the ROI analysis confirmed that the activity of the rostral ACC was indeed negatively correlated with the tinnitus awareness percentage, even after removing other correlated parameters by partial correlation analysis. In other words, the rostral ACC may be the core region of the descending noise cancellation system, and therefore a dysfunction in this area may result in tinnitus perception. Also, the negative correlation between tinnitus awareness percentage and the functional connectivity between the rostral ACC and the left A1 is in line with this concept. This is in line with a recent study revealing reduced functional connectivity between the A1 and subcortical structures in tinnitus sufferers compared to controls [72]. Metabolic activity in the left A1 has been shown to correlate with the presence of tinnitus, independently from the laterality of the perceived tinnitus [73]. Thus the results of this study may designate a dysfunctional top-down cancellation of tinnitus-induced auditory cortical activity by the rostral ACC. The results are in line with a recent neuromodulation study revealing a top-down inhibitory effect on tinnitus via the pg/rostral ACC [15,74]. Also, coupling in the beta-band are believed to be expressed more strongly if the maintenance of the status quo is intended, than if a change is expected [75]. This may further explain why the negative correlation between tinnitus awareness percentage and the functional connectivity between the rostral ACC and the left A1 were found specifically for the beta 1 band. That is, the status quo “no tinnitus” may tend to be changed into tinnitus perception if the noise cancellation system is negatively coupled to the A1.

Unlike aforementioned serial studies [18,20,21,66], we could not find vmPFC on neither source-localized correlation analysis nor ROI-based connectivity analysis. The anatomical difference between the current study and previous studies may be attributed to several factors. First, while previous studies have been performed using structural changes or stimulus-driven hemodynamic changes in small groups of patients, the current study investigated resting-state cortical oscillatory patterns in a large group of patients. Considering that tinnitus is an auditory phantom perception without any externally presented sound stimuli, the findings of the current study, based on resting-state brain electrical activity, may be closer to the pathophysiologic changes in patients with tinnitus. Second, the discrepancy may partly originate from the differences with regard to spatial resolution of the methodologies, i.e., inferior localization ability of quantitative EEG, as compared with MRI. However, considering the small volume (0.125cm^3^) of each ROI in the current study and the distance of the center-of-mass coordinate of vmPFC and that of pg/sgACC (see Table 2), it is less likely that we have misinterpreted vmPFC as pg/sgACC. Nonetheless, future studies using other imaging modalities with high resolution such as magnetic source imaging may further help to confirm the findings of the current study.

### Limitations of the current study

Limitations of the study have to be mentioned. First, although we have collected EEG data of as many as 80 tinnitus participants, the representativeness of EEG data are still limited by the short duration of measurement (five minutes). The most ideal way of measurement may be ambulatory EEG measurement for at least several hours like ambulatory electrocardiography monitoring, but this is not feasible due to technical issues such as difficulties in controlling impedance or shielding against external electrical field. Also, during the measurement, tinnitus may have been present or absent, perhaps more or less strongly so, and we could also assume that the instantaneous effectiveness of the noise cancellation mechanism is possibly related to the instantaneous strength/severity of the tinnitus. Therefore, the identified networks may be involved not in not the immediate generation of tinnitus, but in long-term coping with tinnitus. These limitations may have partially been overcome by recruiting a large number of subjects, but future studies using even larger groups or longer duration of measurement during everyday life, when supported technically, should be performed to replicate the current findings. Second, our methodical strength of a homogeneous group of bilateral NBN tinnitus and normal hearing might have paradoxically facilitated sampling bias. Future studies using subjects with different tinnitus characteristics should be performed to explore if the current findings are generally applicable. Third, recent studies [19,76] have proposed that the tectal longitudinal column (TLC), an auditory equivalent of the periaqueductal gray (PAG) [77], present in all mammals investigated, may be another part of the descending noise cancelling system, based on its distinct connection to the auditory cortex [77] and electrophysiological properties considered to be part of a descending auditory system [78]. However, due to the methodological limitations inherent to EEG, the current study cannot localize subcortical structures such as the TLC. Future studies using fMRI or electrophysiological methods may help in understanding the possible role of the TLC for noise cancellation in tinnitus patients, and thus help in delineating the whole descending noise cancellation pathway. Additionally, neuromodulation studies targeting the rostral ACC should be performed to explore any possible changes in tinnitus awareness percentage by manipulating the activity of the rostral ACC.

## Conclusions

Taken together, the present study reports a negative correlation of the rostral ACC activity and its functional connectivity to the left A1 with the tinnitus awareness percentage in participants with tinnitus. These results may designate the role of the rostral ACC as the core of the descending noise cancellation system, and thus a dysfunctional rostral ACC may result in perception of tinnitus. The present study also suggests the possibility of tinnitus perception modulation by neuromodulatory approaches to change the activity of the rostral ACC.
